# Simultaneous Determination of Four Catechins in Black Tea via NIR Spectroscopy and Feature Wavelength Selection: A Novel Approach

**DOI:** 10.3390/s24113362

**Published:** 2024-05-24

**Authors:** Yabing Liu, Ke Pan, Zhongyin Liu, Yuqiao Dai, Xueyi Duan, Min Wang, Qiang Shen

**Affiliations:** Tea Research Institute, Guizhou Academy of Agricultural Sciences, Guiyang 550025, China; lyb_mdpi@163.com (Y.L.); kepan_gaas@163.com (K.P.); yi2022ying@163.com (Z.L.); dyq777222@163.com (Y.D.); duanxueyigzh2022@163.com (X.D.); minwang_gaas@163.com (M.W.)

**Keywords:** NIRS, black tea, catechin content prediction, wavelength selection, FIC-SS-ELM

## Abstract

As a non-destructive, fast, and cost-effective technique, near-infrared (NIR) spectroscopy has been widely used to determine the content of bioactive components in tea. However, due to the similar chemical structures of various catechins in black tea, the NIR spectra of black tea severely overlap in certain bands, causing nonlinear relationships and reducing analytical accuracy. In addition, the number of NIR spectral wavelengths is much larger than that of the modeled samples, and the small-sample learning problem is rather typical. These issues make the use of NIRS to simultaneously determine black tea catechins challenging. To address the above problems, this study innovatively proposed a wavelength selection algorithm based on feature interval combination sensitivity segmentation (FIC-SS). This algorithm extracts wavelengths at both coarse-grained and fine-grained levels, achieving higher accuracy and stability in feature wavelength extraction. On this basis, the study built four simultaneous prediction models for catechins based on extreme learning machines (ELMs), utilizing their powerful nonlinear learning ability and simple model structure to achieve simultaneous and accurate prediction of catechins. The experimental results showed that for the full spectrum, the ELM model has better prediction performance than the partial least squares model for epicatechin (EC), epicatechin gallate (ECG), epigallocatechin (EGC), and epigallocatechin gallate (EGCG). For the feature wavelengths, our proposed FIC-SS-ELM model enjoys higher prediction performance than ELM models based on other wavelength selection algorithms; it can simultaneously and accurately predict the content of EC ($R_{p}^{2}$ = 0.91, RMSEP = 0.019), ECG ($R_{p}^{2}$ = 0.96, RMSEP = 0.11), EGC ($R_{p}^{2}$ = 0.97, RMSEP = 0.15), and EGCG ($R_{p}^{2}$ = 0.97, RMSEP = 0.35) in black tea. The results of this study provide a new method for the quantitative determination of the bioactive components of black tea.

## 1. Introduction

Black tea is considered one of the most popular beverages among two-thirds of the global population [1,2,3], with unique flavors and health benefits. Currently, consumers have extremely strict requirements for the quality of black tea, which is judged by important indicators such as the content of tea polyphenols and caffeine. Tea polyphenols belong to the polyhydroxy compounds; they are mainly composed of catechins with multiple effects, for example, regulating the gut microbiota, preventing obesity, regulating blood lipids, and antiviral, antibacterial, and antitumor effects [4,5,6]. Caffeine, another important component of tea, can have a stimulating effect on the human body [7]. The contents of tea polyphenols and caffeine vary not only in the variety of tea but also in the leaf positions during the growth of tea trees. By studying the content of tea polyphenols and caffeine in fresh leaves, one can understand the growth status of tea trees in tea gardens and thus manage tea trees more effectively. Meanwhile, this kind of study also provides guidance for selecting leaves with more bioactive components, thus ensuring the high quality of picked tea and of tea industry development. Therefore, there is an urgent need for efficient, non-destructive, and accurate methods of measuring the content of the main active ingredients in fresh black tea, to provide guidance in the rational grading and pricing of tea and the precision management of tea gardens.

The traditional tea quality evaluation is mainly based on artificial senses [8], where professionally trained tea evaluators judge the quality of tea through visual, olfactory, taste, and tactile senses. This requires evaluators to have a high level of professional competence, which cannot meet the needs of online, real-time, and rapid quality monitoring in tea production and distribution. Moreover, their identification results are subjective and less accurate. In recent years, high-performance liquid chromatography (HPLC) has been widely used for the detection of tea polyphenols and other organic matter content in tea [9,10,11]. Unfortunately, these methods not only are expensive, destructive, and slow to analyze, but also require professional operation and cannot be applied to online or portable detection scenarios. As a result, the introduction of an efficient, non-destructive, and cost-effective detection technology for the qualitative and quantitative analysis of black tea’s active ingredients holds significant importance. Near-infrared spectroscopy (NIRS) [12] is a well-established non-destructive testing technique that can be used to describe the high-order and combined vibrational bands of C-H, O-H, and N-H groups in the wavelength range of 780–2500 nm [13,14,15]. More scholars have begun to promote the application of NIRS in tea quality control, and multiple research results have proven that NIRS can achieve accurate quantitative analysis of the main active ingredients in tea.

Ding et al. accurately predicted the content of caffeine and catechin in black and green tea samples by combining NIRS and chemometrics [16]. Luo et al. extracted characteristic wavelengths from complete NIRS wavelengths of green tea through competitive adaptive reweighted sampling (CARS), the successive projection algorithm (SPA), and the random frog method. Then, a tea polyphenol content prediction model was established using partial least squares (PLS), multiple linear regression (MLR), and least squares support vector machine (LS-SVM) with characteristic wavelengths as inputs [17]. Li et al. established an LS-SVM model for the quantitative prediction of eight individual catechins during the black tea drying process, based on near-infrared spectroscopy (NIRS) and chemometric methods such as CARS and SPA [18]. Based on a miniature near-infrared spectrometer, Zou et al. built an SVR model optimized by the grey wolf algorithm to accurately measure the moisture content of black tea, and the prediction accuracy of the nonlinear SVR model was significantly higher than that of the linear PLS model [19]. Ze et al. quickly determined tea polyphenols and other compounds in Pu’er tea using an improved weighted partial least squares algorithm [20]. Chen et al. used the full visible NIR wavelength range of 400–2498 nm, combined with the modified partial least squares regression method, to accurately monitor the concentrations of total catechins and theanine during the tea fermentation process [21]. To quickly detect the content of theanine in oolong tea, Ong et al. proposed a quantitative prediction method using near-infrared spectroscopy combined with a flower pollination algorithm and the Gaussian process regression (GPR) method and achieved good prediction results [22]. These studies have shown the excellent analytical performance of NIRS in predicting the main active components of tea, including catechins, caffeine, theaflavins, and chlorophyll. However, because the multiple monomers of catechin (EC, ECG, EGC, EGCG) contain some identical functional groups in their molecular structures, their near-infrared spectra overlap severely [23], resulting in nonlinear relationships. In addition, the number of near-infrared spectral wavelengths of black tea far exceeds that of modeled samples, resulting in a large amount of redundant information and a typical small-sample learning problem. At present, although some scholars have applied methods such as CARS [17], Monte Carlo uninformative variable elimination (MC-UVE) [24], synergy interval partial least squares [25], and intelligent optimization algorithms [26] to extract effective wavelength features of tea near-infrared spectra, there are usually problems of multiple selection, omission of effective features, or the complexity of a screening process with high randomness.

Therefore, this study proposed an efficient and highly reliable method for screening NIR spectral wavelength features of black tea based on a feature interval combination sensitivity factor and then established a simultaneous prediction model for multiple active ingredients in tea based on a least squares limit learning machine. The main objectives of this study are as follows: (1) to propose an efficient and highly reliable method for extracting the characteristic wavelengths of tea near-infrared spectra most effectively, overcoming the problems of spectral overlap and small-sample learning; (2) to establish a nonlinear and high-precision simultaneous prediction model for the main active ingredients of black tea to solve the problem of spectral nonlinearity.

## 2. Materials and Methods

### 2.1. Black Tea Sample Preparation

The black tea varieties used in this study, including Jinjunmei, Yinghong, and Dianhong, were sourced from different regions like Fujian, Guangdong, and Guizhou provinces in China, during the period from April to July 2022. To achieve sample diversity, fresh tea samples of various varieties were collected from different leaf positions, with a total of 105 selected samples. These samples were subsequently divided randomly, with 70 samples constituting the calibration set and 35 samples the prediction set, in a ratio of 2:1. Before NIRS spectrometer scanning and HPLC analyses were conducted, the tea samples were stored in a refrigerator at 4 ℃ to preserve their integrity. To facilitate further analysis, all samples underwent a grinding process. They were effectively transformed into fine powder using a tube mill (IKA, Breisgau, Germany). Next, the obtained tea powder was used for the subsequent acquisition of near-infrared spectroscopy data and HPLC analyses, which were instrumental in determining the content of catechins and caffeine within each sample, providing reference values for the study.

### 2.2. NIR Spectra Acquisition

This study used a portable spectrometer produced by American Analytical Spectroscopy Equipment Company (ASD), with features such as automatic elimination of dark current and wavelength calibration. Its peak-to-peak signal-to-noise ratio and root-mean-square signal-to-noise ratio measurements in the wavelength range of 4000 to 10,000 cm^−1^ were 0.104 and 0.042, respectively. Each tea sample was placed in a rotatable quartz bottle and then under a spectrometer and was scanned 10 times at a resolution of 4 cm^−1^. The average of 10 measured spectral data was used as a representative of the true near-infrared spectral data of the sample, with a total of 1500 wavelength features in the wavelength range of 4000 to 10,000 cm^−1^. Each wavelength corresponds to the absorbance of black tea (A = log (1/*R*), where *R* is the reflection spectrum). Before the spectral correction model was established, the NIR spectra of all black tea samples were filtered through a Savitzky–Golay (SG) filter [27].

### 2.3. Determination of Catechins and Caffeine Content by HPLC

Following the acquisition of NIR spectral data, 0.1 g of tea powder from each sample was meticulously weighed and placed into a beaker. Next, 20 mL of distilled water was added, and thorough agitation was sustained with a glass rod for 5 min to ensure an even dispersion of the powder within the beaker. The beaker was then heated in a 70 °C water bath for 15 min to facilitate the comprehensive extraction of the primary active components found in tea powder, namely catechins and caffeine. The resulting supernatant was obtained via filtration using a 0.22 μm membrane and subsequently employed for HPLC to measure reference values for catechin and caffeine content. The instrument applied in the measurement process was a Shimadzu LC-2-AD high-performance liquid chromatography system, equipped with a UV–visible light detector. Key measurement parameters including flow rate, detection wavelength, column temperature, injection volume, and the number of tests conducted for each sample were meticulously defined as 1 mL/min, 275 nm, 35 °C, 10 μL, and 5 repetitions, respectively.

### 2.4. Establishment of Quantitative Prediction Models

Due to the similarity in chemical composition of EC, ECG, EGC, and EGCG, their spectral information overlaps severely, resulting in significant nonlinear relationships. The entire spectrum contains 3100 wavelength variables, within which there is a lot of redundant non-feature information. Therefore, it is difficult to achieve efficient and high-precision prediction of tea catechins and caffeine content by establishing just a multivariate linear model for full-spectrum data, which will also affect the generalization ability of the model to varying degrees. Therefore, this study proposed a feature wavelength selection method based on a feature interval combination sensitivity factor and compared it with competitive adaptive reweighted sampling (CARS) [17], Monte Carlo uninformative variable elimination (MC-UVE) [24], and the successive projection algorithm (SPA) [18]. Meanwhile, the study developed a nonlinear quantitative prediction model based on extreme learning machines.

#### 2.4.1. Competitive Adaptive Reweighted Sampling

The CARS is an algorithm that can effectively extract optimal features. Its core principle is to select the most important features for modeling and analysis through competition and adaptive weight allocation. The main process of this method is as follows: first, the wavelength variable with the highest absolute value of the regression coefficient in the model was selected through adaptive reweighting sampling technology, then the wavelength variable with the lowest weight was removed, and finally, the subset with the smallest root-mean-square error (RMSE) was selected through interactive verification.

#### 2.4.2. Monte Carlo Uninformative Variable Elimination

The MC-UVE algorithm evolved from the method of uninformative variable elimination. As a commonly used wavelength screening method, it uses MC simulation to identify uninformative or irrelevant features in the data and eliminate them from the dataset. The core idea of this method is to evaluate the information contribution of features by simulating the randomness in the data, to identify which features are not helpful for problem solving. Although the MC-UVE can quickly remove noise variables, it cannot eliminate a substantial number of non-noise variables with low contribution to the model. This is mainly because the high randomness caused by using the MC algorithm in the sampling process can result in inaccurate calculation of variable importance indicators. Usually, the improvement of accuracy is at the cost of significantly increasing the number of samples, which causes the computational process to be rather time-consuming; this is also the biggest drawback of this method.

#### 2.4.3. Successive Projection Algorithm

The SPA is mainly used to solve the collinearity in multivariate linear regression problems. In recent years, it has been widely used. The SPA achieves data projection and dimensionality reduction by gradually selecting features or variables. It calculates the projection of the selected wavelength on the unselected wavelength each time and introduces the wavelength with the maximum projection distance into the feature wavelength set. Finally, a multiple linear regression model is established for each selected wavelength to obtain the RMSE of the validation set. The feature wavelength subset with the smallest RMSE is the optimal set. The SPA can select wavelengths with the minimum collinearity from all spectral data, thereby reducing redundant information and the number of wavelengths required for the modeling, as well as improving the model performance and interpretability.

#### 2.4.4. Feature Interval Combination Sensitivity Segmentation

The feature interval combination (FIC) method is a new method in response to the severe redundancy of variable information and typical small-sample learning problem in the near-infrared spectrum of black tea. It screens feature wavelength variables and contributes greatly to the model, aiming to improve model accuracy and computational efficiency. This method conducts a comprehensive and segmented screening for the characteristic bands of the main active ingredients of each type of black tea; it avoids the instability of the random sampling process for a single wavelength and reduces the complexity of the wavelength variable screening process. Meanwhile, by using local interval evaluation indicators, it avoids bands with severe spectral overlap of the active ingredients to be tested and screens the characteristic bands of each active ingredient to be tested. The indicators for measuring a certain feature interval variable are RMSE and coefficient of determination (R^2^). The smaller the RMSE, the closer the R^2^ is to 1, and the higher the accuracy of the model. Therefore, by comparing the RMSE and R^2^ values for each sub-interval of the active ingredients to be tested, the optimal set of sub-intervals is selected.

The wavelength screening method of combination feature intervals is used in uniform partitions; however, the characteristic wavelengths of the active ingredients to be tested are not completely distributed in a uniform interval. Therefore, this method may also screen in a small number of wavelengths with low contribution to the model in some selected feature intervals. To solve this problem, it is often necessary to continuously subdivide intervals for better screening of each wavelength. However, this causes both the number of algorithm cycles and the modeling time to increase exponentially. This solution at the cost of time is clearly unscientific.

Therefore, to eliminate the drawbacks of the FIC method, a wavelength selection method based on feature interval combination sensitivity segmentation (FIC-SS) was proposed. This method introduces a sensitivity factor in the original feature interval combination method, which can quickly eliminate the mistakenly selected wavelength variables, thereby achieving higher accuracy in the screening of these variables. The sensitivity factor is defined as the ratio of the absorbance of a certain active ingredient per unit content to the sum of the absorbances of other active ingredients per unit content at a certain wavelength, as expressed in (1):

(1) $$\alpha_{i}=\frac{A_{i}}{\sum_{i=1}^{n}A_{i}},$$

where $A_{i}$ is the absorbance of the *i*-th sample at a certain wavelength. The larger $\alpha_{i}$, the greater the weight of the component in the signal at that wavelength. This means that the component at that wavelength, compared with other ones, has better separation of signals.

The basic steps of this algorithm are as follows:

Step 1: Establish a global PLS model of the ions to be measured within the full-spectrum range, calculate the RMSE and R^2^ values of each ion to be measured according to Equations (7) and (8), and use them as the threshold for feature interval screening.

Step 2: Set *p* cycles, divide the entire spectrum into *p* sub-intervals of equal width, establish a local PLS model for the ions to be tested on each sub-interval, and calculate the RMSE and $R_{i}^{2}$ values for *p* sub-intervals of the active ingredient to be tested.

Step 3: Compare the RMSE and R^2^ values of the full-spectrum model with those of various local models, eliminate wavelength intervals where ${\mathrm{RMSE}}_{i}$ is greater than the global RMSE and $R_{i}^{2}$ is less than the global R^2^, and remove the remaining *q* sub-intervals with smaller RMSE and larger R^2^ values.

Step 4: Combine *t* (1 ≤ *t* ≤ *q*) intervals from the remaining *q* sub-intervals for PLS modeling, and calculate the RMSE and R^2^ values of all possible combination intervals.

Step 5: Use RMSE and R^2^ values as the evaluation criteria for each combination interval, and select the interval combination corresponding to ${\mathrm{RMSE}}_{\min}$ and $R_{\max}^{2}$ as the optimal interval combination.

Step 6: Calculate the α value of all wavelength points in optimal combination interval and the average $\bar{\alpha }$ of each interval for each ion to be tested, according to the sensitivity factor calculation Formula (1). With α as the threshold, eliminate all wavelength points smaller than the average $\bar{\alpha }$ value of each feature interval, and output the final extracted optimal set of feature wavelengths.

#### 2.4.5. Extreme Learning Machine

Extreme learning machine (ELM) [28] is a learning algorithm proposed by Huang et al. in 2004. It aims at effectively training a single-hidden-layer feedforward neural network (SLFN). The input weight and bias of the hidden layer in ELM can be randomly generated, and its hidden layer is the only thing that needs to be determined. In addition to the extremely fast learning speed of ELM, its algorithms also show high generalization performance.

**Figure 1:**
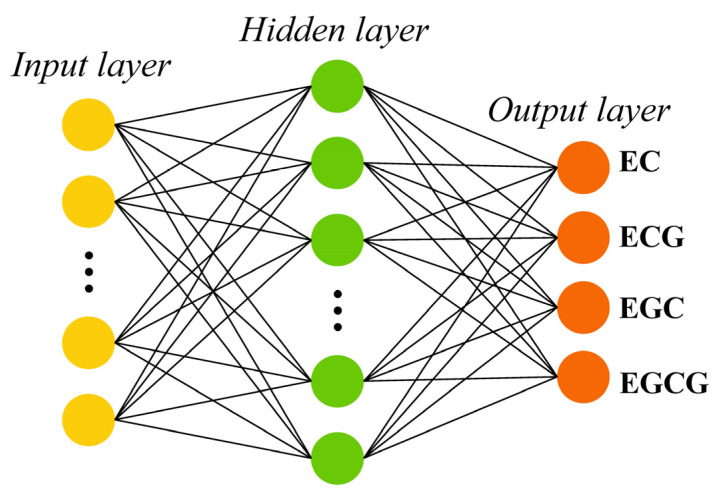
The ELM network structure for simultaneous prediction of four catechins in black tea.

The ELM network structure shown in Figure 1 consists of three layers: input layer, hidden layer, and output layer; each layer contains a different number of neurons. The input layer has *n* neurons; the hidden layer, *l* neurons; and the output layer, *m* neurons. For *n* discrete training samples $\left\{x_{i},c_{i}\right\}$, the input of the model is $x_{i}={\left[x_{i1},x_{i2},\cdots ,x_{in}\right]}^{T}\in {\mathbb{R}}^{n}$, and the output is $c_{i}={\left[c_{i1},c_{i2},c_{i3},c_{i4},c_{i5}\right]}^{T}$. Specifically, the input of the ELM model in this study is the feature wavelength selected by the variable selection algorithm, and the output data of the target are the content of catechins. After training, the ELM model can be obtained as follows:

(2) $$y_{i}=\sum_{j=1}^{l}\beta_{j}g\left(\omega_{j}\cdot x_{i}+b_{j}\right),$$

where $y_{i}={\left[y_{i1},y_{i2},y_{i3},y_{i4}\right]}^{T}$ is the output value predicted by ELM for the input x of the *i*-th training sample. $\beta_{j}={\left[\beta_{j1},\beta_{j2},\cdots ,\beta_{jm}\right]}^{T}\in {\mathbb{R}}^{m}$ and $\omega_{j}={\left[\omega_{j1},\omega_{j2},\cdots ,\omega_{jn}\right]}^{T}\in {\mathbb{R}}^{n}$ are the output weight and the input weight of the jth hidden layer neuron, respectively. $g\left(\cdot \right)$ is the excitation function. As ELM can approach training samples with minimal error, the target output of the training samples can be expressed as follows:

(3) $$c_{i}=\sum_{j=1}^{l}\beta_{j}g\left(\omega_{j}\cdot x_{i}+b_{j}\right),$$

Its matrix form is

(4) C=Hβ,

where $C\in {\mathbb{R}}^{n\times 5}$ is the target output matrix; $H\in {\mathbb{R}}^{n\times l}$ is the hidden layer output matrix, and $\beta \in {\mathbb{R}}^{l\times 5}$ is the hidden layer output weight matrix.

When the hidden layer input weights and offsets are generated, *H* is a constant matrix. Therefore, the β solution can be regarded as solving the least squares special solution problem of a linear system as shown in Equation (5), that is, finding the optimal value to minimize the cost function (the modulus of the difference between the ELM model output and the target output).

(5) $$H\beta^{*}-T=\min_{\beta }\|H\beta -T\|,$$

According to the generalized inverse theory, the least squares special solution $\beta^{*}$ of β is represented as

(6) $$\beta^{*}=H^{†}T,$$

where Moore–Penrose generalized inverse with $H^{†}$ is *H*.

Some parameters of the hidden nodes such as input weights, biases, and impact factors are generated randomly in ELM. Specifically, in each initialization, the weights and biases are uniformly distributed within the range of [−1, 1]. Moore–Penrose (MP) generalized inverse is used to analytically determine the output weights. In our study, the excitation function of ELM was the “sigmoid” function, and the optimum number of hidden nodes for ELM was found by increasing the nodes from 4 to 40 with an increment of 2. The structure with the lowest RMSE was finally selected as the best prediction model. The results were unstable due to random selection of the initial weight values of ELM. Therefore, the test of each model structure was repeated 50 times, and the number of hidden layer nodes was decided by considering the smallest average of iteration results. Finally, the optimum number of hidden nodes was determined to be 14.

### 2.5. Performance Evaluation

The performance of the black tea spectral feature wavelength selection algorithm and the quantitative prediction model was evaluated by determining coefficients, root-mean-square error, and residual prediction bias. Meanwhile, the evaluation indicators for the modeling process were represented by $R_{c}^{2}$ and RMSEC, and the evaluation indicators for the prediction process were represented by $R_{p}^{2}$ and RMSEP. The calculation formulas for R^2^, RMSE, and RPD are as follows [29]:

(7) $$\mathrm{RMSE}=\sqrt{\frac{\sum_{i=1}^{n}{\left({\hat{y}}_{i}-y_{i}\right)}^{2}}{n}},$$

(8) $$R^{2}=1-\frac{\sum_{i=1}^{n}{\left({\hat{y}}_{i}-y_{i}\right)}^{2}}{\sum_{i=1}^{n}{\left({\bar{y}}_{i}-y_{i}\right)}^{2}},$$

(9) $$\mathrm{RPD}=\frac{1}{\sqrt{1-R^{2}}},$$

where *n* is the number of samples, and ${\bar{y}}_{i}$ is the average of the actual chemical values of all samples; $y_{i}$ and ${\hat{y}}_{i}$ represent the measured chemical value and the predicted value of the *i*-th sample, respectively.

## 3. Results and Discussion

### 3.1. Descriptive Statistics of Measured Catechins

**Figure 2:**
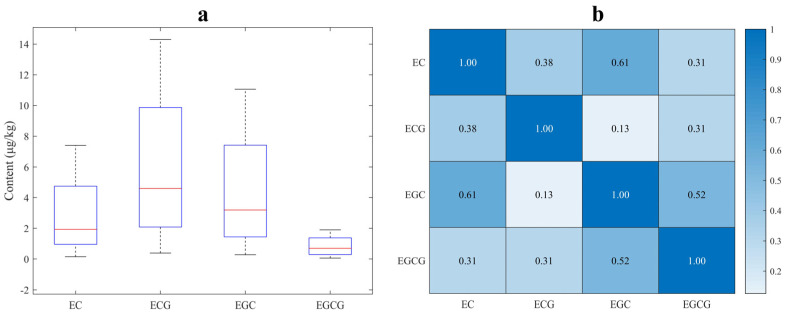
Descriptive statistics of four catechins in black tea. (**a**) Box plot of the EC, ECG, EGC, and EGCG contents; (**b**) correlation analysis between the EC, ECG, EGC, and EGCG contents.

Figure 2a shows the descriptive statistics of the content of EC, ECG, EGC, and EGCG in 87 black tea samples measured by HPLC. The box plot is bounded by 25% and 75% quartiles, with the median values inside; the extreme levels correspond to 5% and 95% quartiles. This figure reveals that the content of ECG in the black tea samples of this study is much higher than that of the other three catechins, confirming ECG as an important active ingredient in fresh tea. Figure 2b shows the Pearson correlation coefficients among the four types of catechins. It can be found that significant positive correlations exist among all catechins except for that between ECG and EGC (r = 0.098), which also demonstrates the overlap and collinearity of their NIRS information.

### 3.2. NIR Spectroscopy Analysis of Black Tea Samples

**Figure 3:**
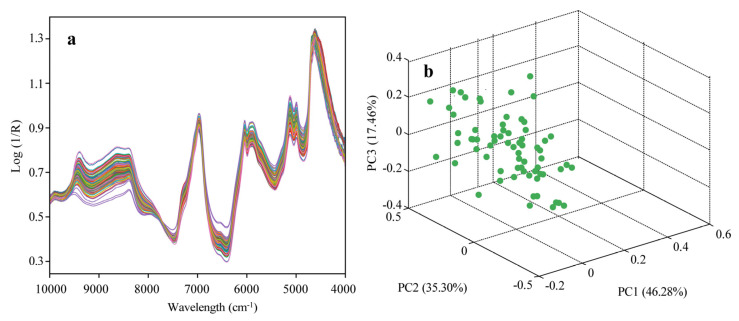
(**a**) Raw spectral curves of black tea samples after SG filter; (**b**) PCA clustering diagram using the first three PCs.

This study utilized a near-infrared spectrometer to acquire spectral absorption data for 87 black tea samples spanning from 4000 to 10,000 cm^−1^. Figure 3a shows the spectral profiles of black tea samples after filtration using the SG method within the 4000 to 10,000 cm^−1^ range. The filtered spectrum, as shown in Figure 3a, displays smooth start and end bands, a notable high signal-to-noise ratio, and an effective filtering outcome, thus satisfying the data quality criteria for quantitative analysis. Moreover, the NIR spectra of black tea exhibit multiple prominent absorption peaks in the regions of 5700–6200 cm^−1^, 6500–7000 cm^−1^, and 8200–8700 cm^−1^. Around these absorption peaks are the pivotal areas of interest for subsequent feature wavelength extraction.

To visualize the spectral distinctions among various black tea samples, a principal component analysis [30] was conducted on the black tea spectra, as demonstrated in Figure 3b. This figure presents the three-dimensional clustering results, where PC1, PC2, and PC3 account for 46.28%, 35.30%, and 17.46% of the variance in spectral variables, respectively. This implies that the first three principal components collectively elucidate 99.04% of the spectral information, disregarding their associations with the various active ingredient contents in tea.

### 3.3. Performance of Prediction Models Based on Full Spectra

To visually demonstrate the phenomenon of low prediction accuracy of linear models caused by the overlap of NIR spectral information in black tea samples, this study conducted a comparative experiment on the prediction performance of ELM and PLS. ELM is a typical nonlinear model, and PLS is the most representative linear chemometric model. Their full-spectrum-based results of the prediction performance evaluation index of four catechins’ content in black tea samples are shown in Table 1. For EGC, the two models share similar prediction performance, with $R_{p}^{2}$ and RPD values greater than 0.8, indicating acceptable prediction accuracy. However, for the other three performance evaluation indices, namely EC, ECG, and EGCG, the PLS model performs significantly worse than the ELM model. This reflects that the ELM model can, to some extent, correct the nonlinearity caused by spectral overlap in black tea samples and thus improve the prediction accuracy of the four catechins’ content. In addition, it can be found that the $R_{p}^{2}$ and RPD predicted for the four catechins’ contents are less than 0.9 and 3.0, respectively, indicating that the predictive performance of the ELM model still needs further optimization. The reasons for the unsatisfactory predictive performance of the ELM model are that there are 1500 spectral variables in the full spectral range of 4000–10,000 cm^−1^, the number of wavelength variables far exceeds the number of modeled samples, and the effective feature wavelength information is submerged. Therefore, extracting feature wavelengths from the full spectrum is an important way to improve the prediction performance of the ELM models.

### 3.4. Performance of Prediction Models Based on Feature Wavelengths

In Section 2.4, we introduced three commonly used spectral feature wavelength selection algorithms, namely CARS, MC-UVE, and SPA, and proposed the FIC-SS algorithm. Therefore, CARS-ELM, MC-UVE-ELM, SPA-ELM, and FIC-SS-ELM models were established to optimize the selection of feature wavelengths most closely related to catechin content, to improve the prediction performance of the ELM model.

#### 3.4.1. FIC-SS-ELM

**Figure 4:**
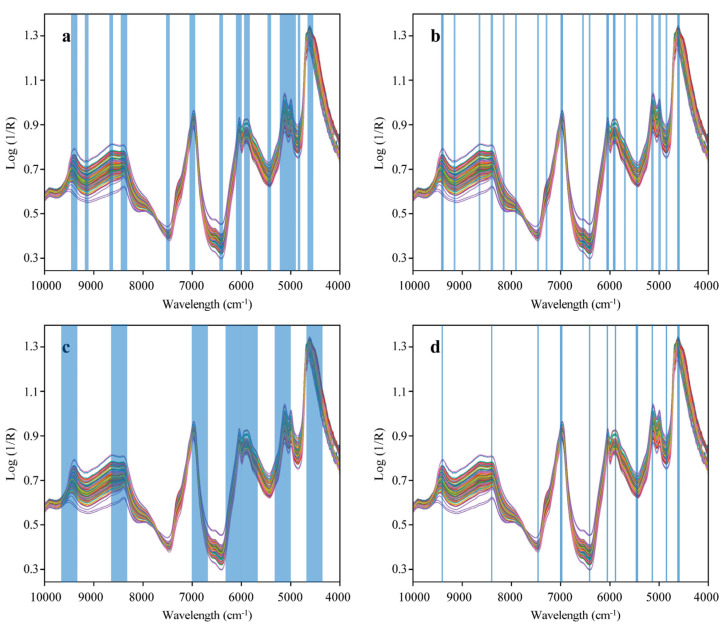
The optimal feature wavelength intervals and variables extracted by four feature wavelength selection algorithms: (**a**) FIC-SS; (**b**) MC-UVE; (**c**) SPA; (**d**) CARS.

The RMSEP and $R_{p}^{2}$ values predicted by the ELM model for EC, ECG, EGC, and EGCG content in Table 1 are used as thresholds for feature interval screening. The full spectrum is divided into 20 sub-intervals, which are modeled by ELM to obtain their RMSEC values. Sub-intervals that fall outside the RMSEC threshold value are eliminated, and the remaining *q* sub-intervals ranging from 2 to *t* (1 < *t* < *q*) are merged to create an ELM model with combination intervals. Sensitivity factors are introduced based on these combination feature intervals, and the average sensitivity factors for each interval within the optimal combination interval are computed as thresholds. Wavelengths with sensitivity factors lower than the average ones are segmented and adjusted. The screening results produced by the FIC-SS algorithm are presented in Figure 4a.

From Figure 4, it is evident that the sensitivity factor segments the optimal combination interval, resulting in varying sub-interval widths. This segmentation effectively eliminates any mistakenly selected wavelengths during the average partitioning of the combination feature intervals and thus enhances the model’s prediction performance. Table 2 presents the prediction performance of the FIC-SS-ELM model. A comparison of Table 1 and Table 2 indicates that FIC-SS-ELM outperforms the ELM model significantly in terms of RMSE, R^2^, and RPD for EC, ECG, EGC, and EGCG content. FIC-SS not only simplifies the model and improves computational efficiency but also, to some extent, mitigates the adverse effects of sample size limitations on prediction performance in the quantitative analysis of black tea using near-infrared spectroscopy.

#### 3.4.2. MC-UVE-ELM

The overlapping spectra of black tea samples were screened according to the MC-UVE process, and the extracted optimal feature wavelength results are shown in Figure 4b. MC-UVE selected a total of 69 feature wavelengths, which can be used to simultaneously predict EC, ECG, EGC, and EGCG content in black tea. The prediction performance of MC-UVE-ELM is shown in Table 2. From Figure 4b, it can be seen that most of the feature wavelengths selected by MC-UVE fall within the range of 8100–8700 cm^−1^. The absorption peaks in this range are caused by the stretching vibration of the second harmonic of C-H, which is the area with the most severe overlap of various active components in tea. The feature wavelengths in this range are not suitable for building the ELM model anymore, as they will have a negative impact on the prediction performance of the model. From Table 2, the prediction performance of MC-UVE-ELM for the four catechins is weaker than that of the FIC-SS-ELM model, but still slightly better than that of the ELM model based on full spectrum.

#### 3.4.3. SPA-ELM

To reduce the complexity of the model, the number of partition intervals for MC-UVE is set to 8 to 20, and the 4 to 8 intervals with the smallest RMSEC are randomly combined as the optimal sub-intervals. Because ELM simultaneously models EC, ECG, EGC, and EGCG, there is no separate feature wavelength screening for the four types of catechins, which can improve the computational efficiency of the model. The extracted optimal sub-interval results of SPA are shown in Figure 4c. From the figure, it can be seen that the optimal number of partitions for SPA is 20, and the optimal sub-intervals selected are those numbered 2, 3, 6, and 10. However, this random combination of intervals brings instability to the prediction performance of the ELM model, resulting in a loss of prediction accuracy. Table 2 shows the prediction performance of SPA-ELM for EC, ECG, EGC, and EGCG, and it is particularly high for ECG.

#### 3.4.4. CARS-ELM

To determine the frequency of variable selection, CARS conducted multiple operations, choosing variables to assess the optimal model results for each catechin across 100 runs. Figure 4d presents 81 selected feature wavelengths using the GARS algorithm. The prediction performance of the CARS-ELM model for EC, ECG, EGC, and EGCG content is documented in Table 2. Table 2 reveals that the CARS-ELM model outperforms the ELM model; however, in the case of EGC, the ELM model exhibits a slightly better RMSEP.

To better demonstrate the performance of different feature selection methods in wavelength selection of black tea NIR spectra, three performance indicators of wavelength selection algorithms, namely the number of feature wavelengths (NFW), the number of wavelength intervals (NWI), and the average wavelength interval width (AWIW) [31], as well as $R_{p}^{2}$, RMSEP, and RPD for these two model prediction performance indicators, were used to comprehensively evaluate the superiority of the FIC-SS algorithm proposed in this paper. An ideal wavelength selection algorithm should balance the number of wavelength intervals and their widths to achieve efficient and accurate prediction outcomes. Choosing the appropriate number of intervals and widths can maximize model performance while controlling model complexity and computational costs.

Table 2 shows that for the four catechins, the FIC-SS-ELM model has the highest prediction accuracy. The SPA method’s AWIW and NWI are significantly higher than those of the other three methods, containing many irrelevant or redundant spectral features, which reduces the ELM model’s processing efficiency and weakens its generalization ability. The CARS method has fewer wavelengths and narrower average wavelength intervals, lacking sufficient spectral information to concurrently describe the four catechins, thus limiting the ELM model’s predictive capability. The FIC-SS method selects 212 wavelength variables, with NWI and AWIW being 13 and 16.31, respectively. This demonstrates that FIC-SS can ensure a larger number of wavelength intervals to include more key spectral information, significantly affecting the target variables while also maintaining a broader wavelength interval width to capture more comprehensive features.

### 3.5. Model Comparison and Discussion

**Figure 5:**
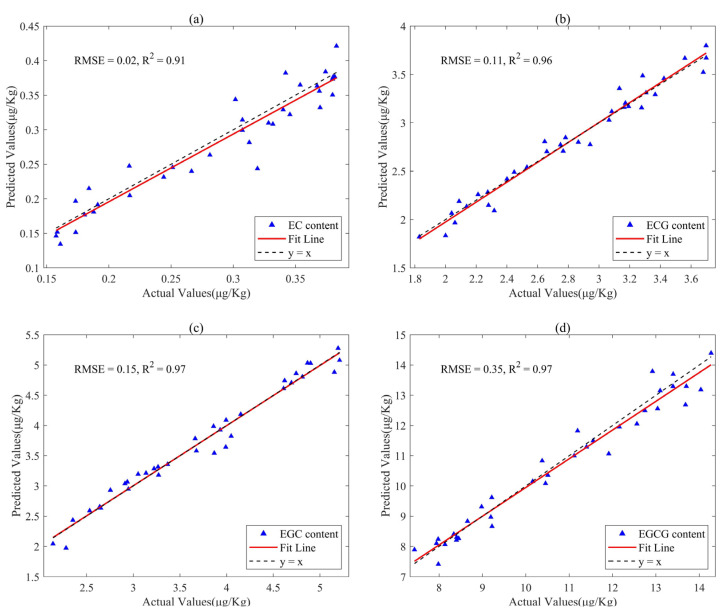
Scatter plots of the FIC-SS-ELM for the prediction of catechins in black tea. (**a**) EC, (**b**) ECG, (**c**) EGC, and (**d**) EGCG content.

Table 1 demonstrates that the ELM model can handle nonlinear relationships in the spectrum of black tea, and can simultaneously determine the content of four catechins in black tea, without the need to repeatedly establish four quantitative prediction models. Through Table 1 and Table 2, it becomes evident that the ELM model founded on feature wavelengths outperforms the ELM model based on the full spectrum in nearly all assessment dimensions and all prediction targets. This highlights that wavelength selection effectively addresses the issue of NIR spectral overlap in black tea and adapts to small-sample quantitative analysis. Figure 5 presents the scatter plots generated by the FIC-SS-ELM model for the prediction of EC, ECG, EGC, and EGCG content. In this figure, the blue circles represent the actual catechin content values of the calibration set of black tea samples measured using HPLC, while the red stars indicate the predicted catechin content values from the model. The figures reveal a striking proximity between the actual values and the model’s predictions, providing an intuitive illustration of the exceptional prediction performance of the FIC-SS-ELM model. The close alignment between the measured values and the predicted ones signifies the model’s robust predictive capabilities, with particularly high accuracy in predicting EGCG content (Figure 5c) and EGCG content (Figure 5d).

By employing four wavelength selection algorithms, this study created four ELM models, and it quantitatively compared their prediction accuracy and robustness using RMSEP and RPD. In Table 2, we observe that for EC content prediction, the accuracy ranks are as follows: FIC-SS-ELM > SPA-ELM = CARS-ELM > MC-UVE-ELM. In terms of robustness, the order is FIC-SS-ELM > SPA-ELM = CARS-ELM > MC-UVE-ELM. The optimal model proposed in this study is FIC-SS-ELM; its prediction performance for EC on RMSEP, R^2^, and RPD values is 0.019, 0.91, and 3.33, respectively. The scatter distributions of the measured and predicted values of the FIC-SS-ELM model for the EC content of the black tea samples are presented in Figure 5a.

For ECG content, the prediction accuracy order is FIC-SS-ELM > SPA-ELM = CARS-ELM > MC-UVE-ELM. Regarding robustness, the ranking is FIC-SS-ELM > SPA-ELM = CARS-ELM > MC-UVE-ELM. As the optimal model proposed in this study, FIC-SS-ELM offers prediction performance for ECG, with RMSEP, R^2^, and RPD values of 0.11, 0.96, and 5.0, respectively. The scatter distributions of the measured and predicted values of the FIC-SS-ELM model for the ECG content of the black tea samples are in Figure 5b.

For EGC content, the prediction accuracy order is FIC-SS-ELM > SPA-ELM = CARS-ELM = MC-UVE-ELM. In terms of robustness, the ranking is FIC-SS-ELM > MC-UVE-ELM > SPA-ELM = CARS-ELM. The optimal model proposed in this study is FIC-SS-ELM, with prediction performance for EGC on RMSEP, R^2^, and RPD values being 0.15, 0.97, and 5.77, respectively. The scatter distributions of the measured and predicted values of the FIC-SS-ELM model for the EGC content of the black tea samples are in Figure 5c.

For EGCG content, the prediction accuracy order is FIC-SS-ELM > CARS-ELM > SPA-ELM > MC-UVE-ELM. As for robustness, the ranking is FIC-SS-ELM = CARS-ELM > MC-UVE-ELM > SPA-ELM. The optimal model is FIC-SS-ELM, whose prediction performance for EGCG on RMSEP, R^2^, and RPD values achieves 0.35, 0.97, and 5.77, respectively. The scatter distributions of the measured and predicted values of the FIC-SS-ELM model for the EGCG content of the black tea samples are in Figure 5d.

The FIC-SS algorithm introduced in this study extracts the most relevant feature wavelengths from the entire black tea spectrum with greater efficiency and stability. This could be attributed to the FIC-SS algorithm’s sequential application of coarse-grained and fine-grained screening during feature wavelength extraction. By combining and refining two or more sub-intervals from the full spectrum via coarse-grained screening, the algorithm identifies the optimal combination interval. Then, the fine-grained screening introduces sensitivity factors of the optimal sub-interval to segment and eliminate mistakenly selected wavelengths. Therefore, the feature wavelengths screened by FIC-SS can build a more robust and accurate model.

## 4. Conclusions

This study brings forth innovation on two fronts: (1) In prior research, present studies mainly applied general wavelength selection algorithms for screening feature wavelengths within the near-infrared spectra of black tea. These algorithms performed single-stage screening, which posed challenges in effectively and reliably extracting feature wavelengths against the substantial spectral overlap observed in black tea catechins. In contrast, this study introduced the FIC-SS wavelength selection algorithm, which is tailored to solving the specific spectral overlap of black tea. With a two-tier screening process, this algorithm operates at both coarse-grained and fine-grained levels, thereby enhancing the accuracy and consistency of feature wavelength extraction. (2) Among the essential active components found in black tea are EC, ECG, EGC, and EGCG. This study developed a nonlinear ELM model based on feature wavelengths, enabling the simultaneous prediction of these four catechin monomers within a unified model. Instead of establishing four separate quantitative prediction models for every single catechin monomer, this approach significantly increased the speed of active ingredient detection in black tea. Furthermore, this study can be a precedent for swiftly assessing the bitterness and astringency intensity of black tea via NIRS.

The experimental results indicated that NIRS technology combined with a wavelength-selection-based ELM model has simultaneously and successfully determined the EC, ECG, EGC, and EGCG content of black tea. In particular, the FIC-SS-ELM model we proposed achieved the best prediction performance, with R^2^ ranging from 0.92 to 0.98 and RPD from 2.6 to 4.8 for these four catechins. This indicated that NIRS combined with chemometrics can accurately determine the bioactive components in black tea and can replace wet chemical analysis. In addition, our proposed method enables rapid online determination of tea quality in tea gardens, and it can also be used for quantitative analysis tasks of other types of spectral techniques.

## Figures and Tables

**Table 1:** The performance of PLS and ELM for the prediction of 4 catechins.

Model	Catechin	Calibration Set		Prediction Set		
		RMSEC	$R_{c}^{2}$	RMSEP	$R_{p}^{2}$	RPD
PLS	EC	0.033	0.75	0.037	0.72	1.89
	ECG	0.25	0.79	0.26	0.76	2.04
	EGC	0.32	0.84	0.34	0.83	2.43
	EGCG	0.76	0.78	0.82	0.77	2.09
ELM	EC	0.027	0.82	0.024	0.81	2.29
	ECG	0.18	0.88	0.21	0.85	2.58
	EGC	0.27	0.87	0.31	0.84	2.50
	EGCG	0.69	0.85	0.73	0.80	2.24

**Table 2:** The performance of the ELM models based on different feature wavelength selection methods for four catechins of black tea.

Catechins	Models	NFW	NWI	AWIW	$R_{p}^{2}$	RMSEP	RPD
EC	FIC-SS-ELM	212	13	16.31	0.91	0.019	3.33
	MC-UVE-ELM	69	19	3.63	0.84	0.025	2.50
	SPA-ELM	325	6	54.17	0.85	0.023	2.58
	CARS-ELM	81	11	7.36	0.85	0.023	2.58
ECG	FIC-SS-ELM	212	13	16.31	0.96	0.11	5.00
	MC-UVE-ELM	69	19	3.63	0.93	0.14	3.78
	SPA-ELM	325	6	54.17	0.94	0.15	4.08
	CARS-ELM	81	11	7.36	0.94	0.15	4.08
EGC	FIC-SS-ELM	212	13	16.31	0.97	0.15	5.77
	MC-UVE-ELM	69	19	3.63	0.94	0.21	4.08
	SPA-ELM	325	6	54.17	0.93	0.21	3.78
	CARS-ELM	81	11	7.36	0.93	0.21	3.78
EGCG	FIC-SS-ELM	212	13	16.31	0.97	0.35	5.77
	MC-UVE-ELM	69	19	3.63	0.94	0.45	4.08
	SPA-ELM	325	6	54.17	0.92	0.53	3.54
	CARS-ELM	81	11	7.36	0.97	0.35	5.77

## Data Availability

The data that support the findings of this study are available upon request from the corresponding author. The data are not publicly available due to privacy or ethical restrictions.
